# Reducing the burden of tobacco: what’s the endgame?

**DOI:** 10.1186/2045-4015-3-36

**Published:** 2014-10-28

**Authors:** Michael Chaiton, Robert Schwartz

**Affiliations:** Dalla Lana School of Public Health, University of Toronto, Ontario Tobacco Research Unit, 155 College St, Toronto, Ontario M5T 3M7 Canada

## Abstract

Tobacco use causes a tremendous amount of morbidity and mortality globally, with a staggering level of financial costs. In many countries, public health interventions have been able to reduce the prevalence of smoking and the associated burden. However, despite these successes, there is still much work left to be done. This commentary argues that the tobacco control interventions recommended by the World Health Organization are necessary but not sufficient to adequately address the consequences of tobacco use.

The campaign against tobacco has been one of the most successful public health endeavors in modern history. Much of the success in tobacco control is attributable to regulations and policies that have addressed public education, economic incentives to use tobacco, and protection of non-smokers. Prevalence rates have fallen by half in many countries, resulting in potentially hundreds of millions of lives saved worldwide.

Yet, the magnitude and scope of the tobacco epidemic remains massive. Smoking causes a level of burden that would not be acceptable from any other commercial product. Ginsburg and Geva estimate, conservatively, that in 2014 alone, nearly 8000 deaths could be attributed to smoking tobacco in Israel [1]. The good news is that this estimate is down from close to 11,000 deaths attributable to tobacco in 2001. Even with ignoring population growth, the decline in smoking prevalence has protected 60 people from tobacco use related death each and every week, in Israel alone. This account does not even take into consideration the suffering that tobacco use causes to many people from chronic diseases, various infectious diseases and from longer recovery from certain surgical procedures. Furthermore, the lag between tobacco exposure and the onset of disease means that the burden will continue to decrease into the future, purely based on changes in prevalence that have already occurred.

Ginsburg and Geva also highlight that the costs associated with smoking go beyond the price of mortality and morbidity to include a substantial financial burden. Their cost estimate is once again a conservative approximation of the total financial burden, focusing on direct health care costs. It only roughly estimates the costs due to abstenteeism and leaves aside private costs due to smoking breaks, illness and loss of productivity due to years of productive life lost. Philip Morris, among others, suggests cynically that early mortality is a benefit to government coffers [2], but ignores the substantial death and disability that occurs among those of working age. It is well known that cigarette smokers live 10 years less on average [3], but it is less known that a male nonsmoker has an 81% chance of living to 70 years old, while a male smoker has only a 55% chance of making it to that milestone alive.

As Ginsburg and Geva report, globally, it is estimated that 5 million deaths each year are attributable to smoking, with trends driving a rise to as much as 10 million deaths per year by the 2030s [4]. In response, the World Health Organization [WHO] has set out the Framework Convention for Tobacco Control [FCTC]. The FCTC and its guidelines provide the foundation for countries and health regions to implement and manage tobacco control [5]. To help make this a reality, the MPOWER package of measures was introduced by WHO in 2008. The MPOWER Report [6] has defined a set of policies that are consistent with the FCTC, which includes Monitor tobacco use and prevention policies, Protect people from tobacco smoke, Offer help to quit tobacco use, Warn about the dangers of tobacco, Enforce bans on tobacco advertising, promotion and sponsorship, and Raise taxes on tobacco. MPOWER guidelines require that each nation imposes taxes on cigarettes that constitute 75% of the retail price; implements comprehensive smoke-free indoor air laws and advertising/marketing restrictions; requires large, bold and graphic health warnings; provides broad access to cessation treatments; and implements well-funded tobacco control media campaigns.

David Levy’s SIMSMOKE model has been used to calculate the expected change in smoking attributable mortality in Israel, if various interventions are adopted [7]. This SIMSMOKE model [using the Doll et al. finding that half of long term users are killed by tobacco] projects that due to policy changes between 2007 and 2010, 35,264 deaths will be averted in the future due to offering cessation treatments and 80,134 will be averted due to tax increases.

In Ontario, Canada—our home province-- we have applied the SIMSMOKE model to a jurisdiction with relatively strong tobacco control policies [8]. We found that implementation of the full package of MPOWER suggestions to the highest level could reduce current smoking prevalence from an already low 18% in 2013 to 12% in 2043, and save 50,000 lives over that period. These results suggest that these well studied and effective interventions should be implemented immediately for greatest effect.

On the other hand, the results from Ontario are disappointing—despite the implementation of a strong package of tobacco control policies over a period of thirty years (and nearly 90 years after the acceptance of the health risks associated with smoking) optimistic projections find that more than 1 in 10 people will continue to smoke. This suggests that much bolder and more effective policies are needed to bring tobacco use and the burden associated with tobacco use down to acceptable levels in Canada, in Israel, and in other jurisdictions around the world. That is, while the MPOWER package will have a substantial impact on public health, more comprehensive policies will be needed to reduce the prevalence of smoking further and faster.

There are several ‘beyond MPOWER’ policy measures that are supported by some evidence. Jha and Peto recommend at least of tripling of the taxes on tobacco to make a substantial dent in the use of tobacco [9]. Plain packaging and large graphic warnings could be a strong policy tools to reduce the number of adolescents starting smoking [10]. Smoking bans in more public places, including patios, doorways and parks, could not only decrease physical and social exposure to tobacco use, but also increase cessation and decrease initiation [11]. Efforts to control the rampant availability of tobacco on every corner may help smokers stay quit, encourage additional quit attempts and decrease initiation [12]. Similarly, product regulations could make smoking less appealing to novice and experienced smokers.

Policy makers will also have to deal with new challenges like tobacco promotion via social media and the use of e-cigarettes [13]. There are no guarantees that tobacco use will continue to decline into the future without close attention to the issue and further action from those interested in public health. Politicians and health policy advisors consistently underestimate public preference for bold tobacco control action [14]. Now is the time to start thinking about the endgame for tobacco and the policies and regulations that will reduce the burden of tobacco to zero. While we have much to celebrate in terms of the successes in tobacco control, Ginsburg and Geva remind us of the tremendous burden still affecting Israel and countries around the world.

## Author information

Michael Chaiton is a Scientist at the Ontario Tobacco Research Unit and Assistant Professor at the Dalla Lana School of Public Health, University of Toronto. He has a PhD in Epidemiology from the University of Toronto. He is the co-instructor of the graduate level course “Tobacco and Health: From Cells to Society” and is Co-Head of the Population Research Initiative in Mental Health and Addictions (PRIMHA) at OTRU. His main areas of research include smoking cessation, smoking co morbidities, and understanding the impact of widespread retail availability of tobacco. He holds a career development award from the Canadian Cancer Society.

Robert Schwartz is the Executive Director of the Ontario Tobacco Research Unit. He is an Associate Professor at the Dalla Lana School of Public Health, University of Toronto, Senior Scientist at the Centre for Addiction and Mental Health and holds a faculty association at the University’s school of Public Policy and Governance. Rob directs the CIHR Strategic Training Program in Public Health Policy and is Editor-in-Chief of the Canadian Journal of Program Evaluation. He currently teaches two courses in public health policy and has taught several courses in evaluation, budgeting, and performance measurement and analysis. Dr. Schwartz has published widely in the areas of tobacco control, public health policy, evaluation, accountability, and policy change.

## Commentary on

Ginsberg GM, Geva H: The burden of smoking in Israel–attributable mortality and costs (2014). Israel Journal of Health Policy Research. 2014 Aug 29;3:28. doi:10.1186/2045-4015-3-28.
